# Probability of severe postpartum hemorrhage in repeat cesarean deliveries: a multicenter retrospective study in China

**DOI:** 10.1038/s41598-021-87830-7

**Published:** 2021-04-19

**Authors:** Lili Du, Ling Feng, Shilei Bi, Lizi Zhang, Jingman Tang, Liuying Zhong, Xingnan Zhou, Hu Tan, Lijun Huang, Lin Lin, Shanshan Zeng, Luwen Ren, Yinli Cao, Jinping Jia, Xianlan Zhao, Shaoshuai Wang, Xiaoyan Xu, Yangyu Zhao, Zhijian Wang, Qiying Zhu, Hongbo Qi, Lanzhen Zhang, Suiwen Wen, Hongtian Li, Jingsi Chen, Dunjin Chen

**Affiliations:** 1 Department of Obstetrics and Gynecology, Key Laboratory for Major Obstetric Diseases of Guangdong Province,The Third Affiliated Hospital of Guangzhou Medical University, 63 Duobao Road, Guangzhou, 510150 Guangdong China; 2 Key Laboratory for Reproduction and Genetics of Guangdong Higher Education Institutes, Guangzhou, China; 3 Department of Obstetrics and Gynecology, Northwest Women’s and Children’s Hospital, Xi’an, China; 4 Department of Obstetrics and Gynecology, Guangzhou Huadu District Maternal and Child Health Hospital, Guangzhou, China; 5 Department of Obstetrics and Gynecology, The First Affiliated Hospital of Zhengzhou University, Zhengzhou, China; 6 Department of Obstetrics and Gynecology, Tongji Hospital, Tongji Medical College, Huazhong University of Science and Technology, Wuhan, China; 7 Department of Obstetrics and Gynecology, Peking University Third Hospital, Beijing, China; 8 Department of Obstetrics and Gynecology, Nanfang Hospital, Southern Medical University, Guangzhou, China; 9 Department of Obstetrics and Gynecology, The First Affiliated Hospital of Xinjiang Medical University, Ürümqi, China; 10 Department of Obstetrics and Gynecology, The First Affiliated Hospital of Chongqing Medical University, Chongqing, China; 11 Department of Obstetrics and Gynecology, The Second Affiliated Hospital of Guangzhou Medical University, Guangzhou, China; 12 Department of Obstetrics and Gynecology, The Sixth Affiliated Hospital of Guangzhou Medical University, Qingyuan People’s Hospital, Guangzhou, China; 13 Institute of Reproductive and Child Health, National Health Commission Key Laboratory of Reproductive Health, Peking University Health Science Center, Beijing, China

**Keywords:** Diseases, Risk factors

## Abstract

To determine the factors predicting the probability of severe postpartum hemorrhage (SPPH) in women undergoing repeat cesarean delivery (RCD). This multicenter, retrospective cohort study involved women who underwent RCD from January 2017 to December 2017, in 11 public tertiary hospitals within 7 provinces of China. The all-variables model and the multivariable logistic regression model (pre-operative, operative and simple model) were developed to estimate the probability of SPPH in development data and external validated in validation data. Discrimination and calibration were evaluated and clinical impact was determined by decision curve analysis. The study consisted of 11,074 women undergoing RCD. 278 (2.5%) women experienced SPPH. The pre-operative simple model including 9 pre-operative features, the operative simple model including 4 pre-operative and 2 intraoperative features and simple model including only 4 closely related pre-operative features showed AUC 0.888, 0.864 and 0.858 in development data and 0.921, 0.928 and 0.925 in validation data, respectively. Nomograms were developed based on predictive models for SPPH. Predictive tools based on clinical characteristics can be used to estimate the probability of SPPH in patients undergoing RCD and help to allow better preparation and management of these patients by using a multidisciplinary approach of cesarean delivery for obstetrician.

## Introduction

The increasing rates of cesarean delivery (CD) worldwide are a growing public concern^1,2^. In China, the rates of CD increased from 28.8% in 2008 to 34.9% in 2014 and reached 36.7% in 2018^3^. Consequently, there has been a significant increase in the number of women who have had repeat cesarean deliveries (RCDs) worldwide. In the US, the rate of RCDs increased by 178% from 5.2% in 1979 to 14.4% in 2010^4^. Recently, a cross-sectional study found that the rate of CD in Shanghai, China, was 41.5% in 2016, and the majority of women with a previous CD had an RCD (96.6%)^5^. Compared with a normal birth and the first CD, RCDs are associated with increased maternal and neonatal morbidity^6,7^.

Severe postpartum hemorrhage (SPPH) is an important cause of maternal death and severe maternal morbidity^8^. Compared with vaginal delivery and primary CD, women undergoing RCD are at a higher risk of postpartum hemorrhage (PPH) and hemorrhage-related morbidity^9^. Identification of the risk factors for SPPH and development of a clinical predictive model could optimize patient outcomes by improving the planning and timely mobilization of staffing and resources before the onset of bleeding and by enhancing postpartum surveillance for excessive blood loss in case of women with high-risk pregnancies.

Several studies have consistently shown that women who undergo RCDs are at increased risk of dense adhesions and abnormal placentation^10,11^. The timing of delivery and the number of CDs are associated with increasing adverse maternal outcomes^6,12^. However, whether other risk factors for SPPH exist in patients with RCDs is unknown. In addition, although several risk assessment tools have been created to predict women at risk for PPH, Kawakita et al. found that these tools had a moderate predictive power to identify women at a high risk for SPPH after CD^13^. Therefore, separate risk assessment tools based on the mode of delivery, especially for patients with RCDs, should be developed.

This study aimed to evaluate various clinical features and ultrasound risk factors that predict SPPH in patients with RCD. It also sought to establish an effective model to facilitate the prediction of SPPH in women undergoing RCD to allow better preparation and management of these patients by using a multidisciplinary approach of CDs.

## Results

### Characteristics of the pregnant women and maternal and neonatal outcomes in SPPH and non-SPPH group

**Figure 1:**
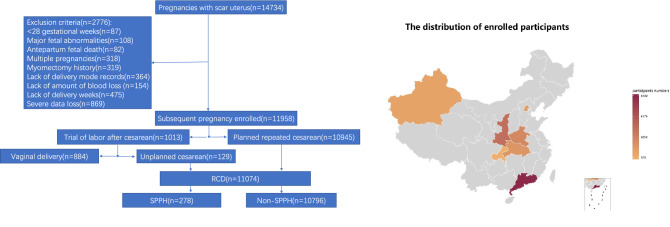
Cohort flowchart and distribution of study population. The map was created by R software (version 3.6.1). URL link: http://cos.name/wp-content/uploads/2009/07/chinaprovinceborderdata_tar_gz.zip. *SPPH* severe postpartum hemorrhage, *RCD* repeat cesarean delivery.

The study consisted of 11,074 singleton women with a history of CD who underwent RCD. Of these pregnancies, 98.8% (10,945/11,074) underwent elective RCD and 1.2% (129/11,074) with failure of vaginal birth after cesarean (VBAC). In total, 278 women experienced a cumulative blood loss of 1500 mL or more, and 10,796 women experienced blood loss less than 1500 ml (Fig. 1).

Supplementary Table S1 online showed all the characteristics of the pregnant women which includes 38 pre-operative and 2 operative variables. The incidence rate of adverse maternal outcomes including blood transfusion, bladder injury, hysterectomy, infection and disseminated intravascular coagulation (DIC) was higher in SPPH group compared with non-SPPH group. More newborns were with complications and need therapies in SPPH group (Table 1).

**Table 1:** Maternal and neonatal outcomes in SPPH and non-SPPH group.

Variables	Non-SPPH (n = 10,796)	SPPH (n = 278)	*P*
Blood transfusion	376(3.5%)	183(65.8%)	< 0.05
Bladder injury	157(1.5%)	32(11.5%)	< 0.05
Part or complete hysterectomy	1(0)	16(5.8%)	< 0.05
Infection	41(0.4%)	4(1.4%)	< 0.05
DIC	1(0)	6(2.2%)	< 0.05
Neonatal therapy	944(8.7%)	83(29.9%)	< 0.05
Neonatal complications	271(2.5%)	35(12.6%)	< 0.05

*SPPH* severe postpartum hemorrhage, *DIC* disseminated intravascular coagulation.

### Four risk models to predict SPPH

Table 2 summarized the most significant features included in the three simple models described above: the pre-operative simple model including 9 pre-operative variables, the operative simple model including 4 pre-operative and 2 intraoperative variables and the simple model including 4 pre-operative variables.

**Table 2:** Significant variables included in the three simple models.

Variables	Pre-operative simple model		Operative simple model		Simple model	
	OR (95%CI)	*P*	OR (95%CI)	*P*	OR (95%CI)	*P*
PP	10.427(6.892–15.777)	***	6.370(4.270–9.503)	***	6.6114(4.427–9.880)	***
Placenta accreta	6.407(4.416–9.295)	***	5.580(3.904–7.975)	***	6.337(4.435–9.055)	***
Gestational weeks	0.864(0.810–0.921)	***	0.863(0.815–0.915)	***	0.844(0.798–0.892)	***
Source	0.476(0.348–0.650)	***				
Oligohydramnios	0.203(0.118–0.349)	***				
Endometrial injury	2.417(1.370–4.267)	**	2.562(1.481–4.430)	**	2.460(1.420–4.262)	**
Numbers of CD	1.565(1.134–2.159)	**				
Interval months	1.005(1.001–1.008)	**				
Time of RCD	0.680(0.487–0.949)	*				
Pelvic adhesion during RCD			1.980(1.513–2.592)	***		
Uterine incision during RCD			1.631(1.281–2.077)	***		

*PP* placenta previa, *CD* cesarean delivery, *RCD* repeated cesarean delivery, *OR* odds ratio, *CI* confidence interval. ***0.001 **0.01 *0.05.

Endometrial injury other than history of prior cesarean delivery includes uterine curettage, hysteromyomectomy, hysteroscopic surgery and other reason that damaged the endometrium; Source includes patients in hospital and referral; Time of RCD includes elective and emergency CD; Uterine incision status during RCD includes normal, thin incision, incomplete and complete rupture.

**Figure 2:**
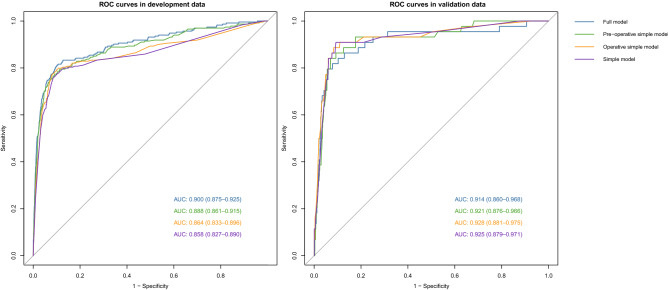
ROC curves of the four models in the development and validation data. *ROC curve* receiver operating characteristic curve, *AUC* area under the receiver operating characteristic curve.

**Figure 3:**
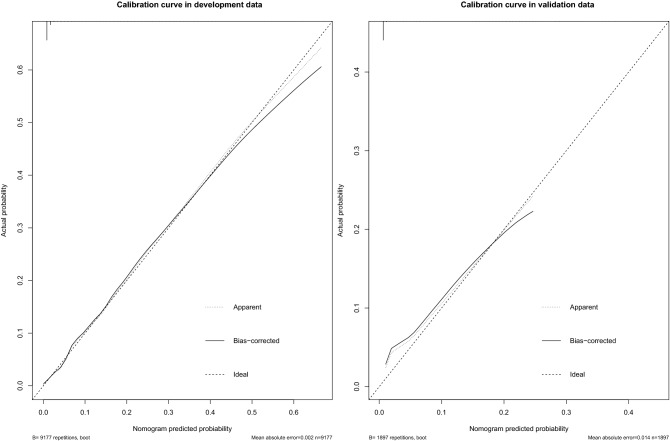
The calibration curve of the simple model for the probability of SPPH in the development and validation data.

Figure 2 showed the excellent discrimination of the four models in the development and validation data. The area under the receiver operating characteristic curves (AUC) using the full model, pre-operative simple model, operative simple model and simple model were 0.900, 0.888, 0.864 and 0.858, respectively in the development data and 0.914, 0.921, 0.928 and 0.925 in the validation data. The calibration curve of the simple model for the probability of SPPH demonstrated good agreement between prediction and observation in the development and validation data (Fig. 3). *P* values in Hosmer–Lemeshow test of the simple model were 0.865, and 0.112 in the development and validation data. In the development and validation datasets, simple model predicted probabilities of SPPH showed good separation between participants in whom SPPH was and was not diagnosed (see Supplementary Fig. S1) online).

Table 3 showed the way in which the prediction accuracies of the four models varied according to risk cutoff points for defining a positive screening result. For example, if a threshold of at least a 5% risk of SPPH was used with the simple model, the sensitivity, specificity, positive predictive value, and negative predictive value were 84.1%, 92.9%, 21.9%, and 99.6%, respectively.

**Table 3:** Prediction accuracies of the four models varied according to risk cutoff points for defining a positive screening result.

Models	Risk score threshold (%)	Sensitivity (%)	Specificity (%)	PPV (%)	NPV (%)	Accuracy (%)	Detection prevalence (%)	Balanced accuracy (%)
Full	≥ 2	0.841	0.895	0.16	0.996	0.894	0.122	0.868
	≥ 5	0.795	0.928	0.207	0.995	0.925	0.089	0.862
	≥ 10	0.75	0.943	0.237	0.994	0.938	0.073	0.846
Pre-operative	≥ 2	0.864	0.9	0.17	0.996	0.899	0.118	0.882
	≥ 5	0.841	0.926	0.211	0.996	0.924	0.092	0.883
	≥ 10	0.795	0.94	0.24	0.995	0.937	0.077	0.868
Operative	≥ 2	0.909	0.883	0.156	0.998	0.884	0.135	0.896
	≥ 5%	0.864	0.922	0.208	0.997	0.92	0.096	0.893
	≥ 10%	0.841	0.938	0.243	0.996	0.936	0.08	0.889
Simple	≥ 2%	0.909	0.877	0.149	0.998	0.878	0.141	0.893
	≥ 5%	0.841	0.929	0.219	0.996	0.927	0.089	0.885
	≥ 10%	0.795	0.94	0.238	0.995	0.936	0.077	0.868

*PPV* positive predictive value, *NPV* negative predictive value.

**Figure 4:**
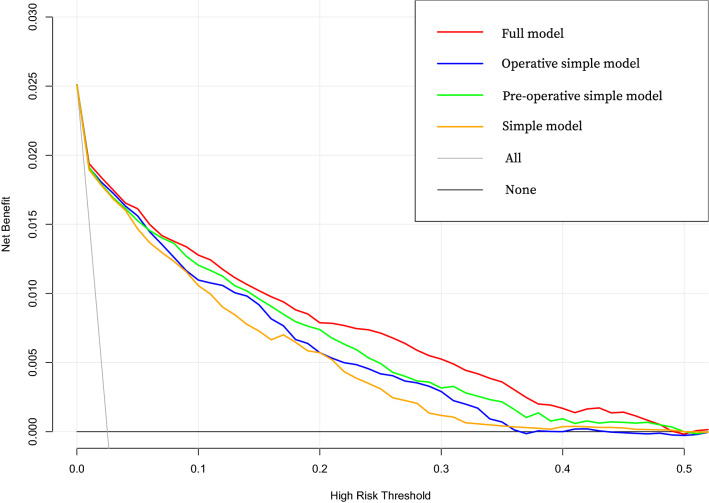
DCA of the four models in all data. DCA shows the clinical usefulness of four models based on a continuum of potential thresholds for SPPH (x-axis) and the net benefit of using the model to risk stratify patients (y-axis) relative to assuming that no patient will have SPPH. *DCA* decision curve analysis.

In Fig. 4 Decision curve analysis (DCA) graphically showed the net benefit across the range of SPPH risk threshold of the four models in all data. DCA of the four models in development data and validation data were showed in Supplementary Fig. S2.

**Figure 5:**
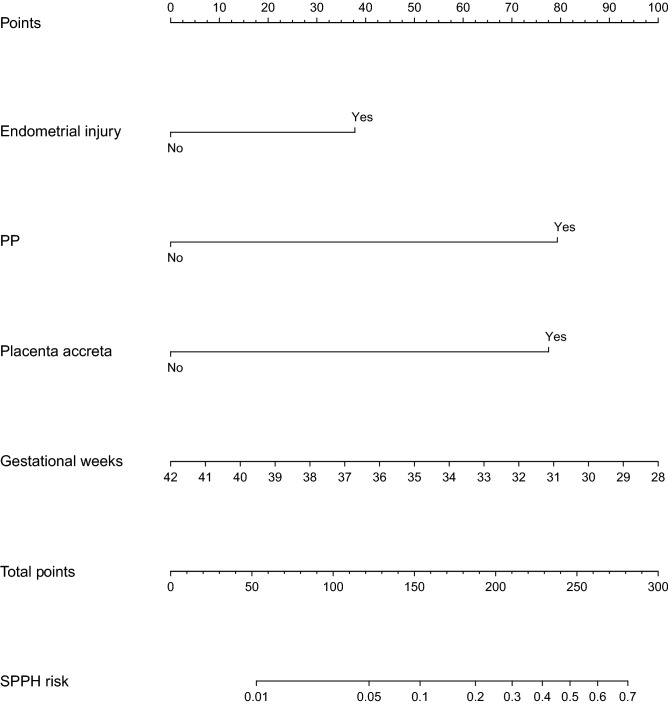
Nomogram of simple model. *PP* placenta previa, *SPPH* severe postpartum hemorrhage.

The pre-operative simple model and simple model were presented as a graphical calculators (nomogram) in Supplementary Fig. S3 online and Fig. 5 to predict SPPH. The nomogram converted each risk predictor into a 0–100 scale that was proportional to the derived adjusted log odds. These points were added across predictors to derive the ‘‘total points’’, which were converted to predict the probabilities of SPPH. This nomogram facilitated the calculation of the probability of SPPH for obstetricians.

## Discussion

In this large-scale multicenter study, the prevalence of SPPH was 2.5% (278/11,074). The effect of different factors (40 variables) on the risk of SPPH in patients with RCD was evaluated. We found that six factors, namely endometrial injury other than history of prior cesarean delivery, placenta previa (PP), placenta accreta, gestational weeks, pelvic adhesion, and uterine incision status independently influenced the occurrence of SPPH in RCD patients. Considering that pelvic adhesion and previous uterine incision status are intraoperative variables, it was a little late to predict SPPH. Of the pre-operative factors (38 variables), we found that nine factors, namely endometrial injury other than history of prior cesarean delivery, PP, placenta accreta, gestational weeks, oligohydramnios, numbers and time of CD, interval months, and source of patients were related with SPPH patients. We found that there were four variables which were closely associated with SPPH both in pre-operative and operative simple models. They were endometrial injury other than history of prior cesarean delivery, PP, placenta accreta, and gestational weeks.

In this study, SPPH was defined as blood loss exceeding 1500 mL. Although this definition varied between the guidelines of different countries, special measures should be implemented to save the patient’s life when postpartum blood loss of over 1,500 mL occurs, according to the guideline of prevention and treatment of PPH published by the Department of Gynecology and Obstetrics of the Chinese Medical Association^14^. It was reported that compared with women without SPPH, those with SPPH had 115 times, 86 times, and 3.5 times the risk of hysterectomy, acute renal failure, and sepsis, respectively, along with increased frequency of admission to the intensive care unit (ICU), reflecting the severity and potential lethality of this complication^15^. In our multicenter cohort, among the 278 women who underwent RCDs and experienced SPPH, 183 (66%) required blood transfusion, bladders of 32(12%) patients were damaged, 16 (6%) required a hysterectomy, 4 (1%) and 6 (2%) patients experienced infection and DIC. Hence, it was important to establish a predictive model to accurately evaluate the risk of SPPH in patients with RCDs in order to help clinicians to plan and implement measures required for women identified to be at high risk for developing SPPH.

Several models such as CMQCC, AWHONN, and NYSBOH were reported to predict PPH in women who underwent vaginal and cesarean deliveries. Kawakita et al. evaluated and compared the validity of these three risk assessment tools to predict SPPH in women undergoing CDs at or beyond 23 weeks of gestation^13^. Kawakita et al.’s study found that these three available tools had a moderate predictive power for predicting patients who would require CD and stated that future studies should focus on the development of separate risk assessment tools based on the mode of delivery.

In our study, we developed and validated four models that used ascertained clinical features for comprehensive and accurate risk estimation of SPPH in patients with RCD. The pre-operative and operative simple model were used to predict SPPH at different time. Furthermore, the simple model used the fewer clinical features which was also excellent with AUC > 0.9 in the validation cohort and good goodness of fit. We also used Decision Curve Analysis (DCA) to evaluate the models. The decision curve of a model is compared to extreme cases that including all patients or none. A model can be recommended for clinical use if its net benefit is greater than treating all and none patients. Though the probability range of patients’ benefit is small, three simple models were better than extreme situations (None and All) in development data and validation data. Out study validated the ability of the models to predict SPPH in repeat cesarean deliveries. A larger cohort study is needed to validate the feasibility of applying such models in clinical practice further.

Coupled with the increasing rate of CDs worldwide, the incidence of placenta previa and accreta also significantly increased^16^. Endometrial injury has been reported to lead to abnormal placental implantation (placenta previa and accreta)^17^. The placenta in the lower uterine segment enlarges and invades deeper to ensure blood supply^18^. The muscular layer around the scar area is lacking or intermittent, which influences the contraction of the uterine muscles and the compression of blood vessels. This could account for the fact that a thin uterine segment is a risk factor for SPPH. Further, the abnormally invasive placenta could lead to life-threatening hemorrhage as placental separation proceeds. The American College of Obstetricians and Gynecologists and International Federation of Gynecology and Obstetrics (FIGO) guidelines recommend that blood transfusions and hysterectomy should be considered in women with placenta previa and placenta accreta^19,20^. In line with these studies, intraperitoneal adhesions caused by previous CDs would prolong the operation time, cause severe bleeding, necessitate hysterectomy, and cause bladder and intestinal injury, and even acute morbidity in patients with RCDs^7,11^. Lower gestational age at RCD was also a risk factor for SPPH. Breslin et al. found that the incidence of adverse maternal outcomes increased with delivery before 39 weeks of gestation in women with three previous CDs^6^. In our cohort data, the mean gestational age at delivery was lower in the SPPH group.

This study showed that incorporating predictive models into obstetric clinical practice might be feasible. In our study we found that, oligohydramnios was less in SPPH group than non-SPPH group. Sarah Crimmins reported that polyhydramnios increased the risk of PPH^21^ and Lester Figueroa et al. reported that oligohydramnios was the risk factor of PPH^22^. We also found that with the interval between two CDs more than 4 years, the longer the time was, the greater the possibility of SPPH was. In our study the incidence rate of SPPH was higher in referral RCD patients but lower in emergency RCD patients. Future larger and prospective trials should be investigated thoroughly to help obstetricians better understand the patients’ characteristics in the predictive models. Additionally, real-time modulation of predictive models is needed depending on patients conditions.

Our study had several strengths. This was a large-scale multicenter study that involved a detailed assessment of a large sample of patients with RCDs from different districts of China for risk of SPPH. All data are of high quality, as they were obtained by chart review. From 40 variables, 4 factors were identified as important factors to develop a simple model for risk assessment of SPPH in patients with RCD; this model had been demonstrated in the validation dataset and in the separate hospital datasets (see Supplementary Fig. S4 online). However, the study has some limitations. This model was developed from dataset which consisted of singleton women with a history of CD who underwent RCD. It was uncertain whether it was effective to predict SPPH risk in patients with twin RCDs. As the factors associated with postpartum hemorrhage in twin pregnancies seemed to be different from those in singleton pregnancies^23^, a new model specifically for twin RCDs may need to be developed in future study. We were unable to further classify placenta previa as marginal, partial and complete placenta previa. At the same time, placenta adhesion, placenta accreta and penetrating placenta accreta should be studied respectively. This information was not available in some hospitals’ datasets, and thus we were unable to develop a more accurate prediction model including different classification of placenta previa and placenta accreta. However, based on our previous 10-year retrospective cohort data from Third Affiliated Hospital of Guangzhou Medical University and Tongji Hospital, complete placenta previa was a risk factor of PPH^24^.

## Conclusions

In this study, we developed nomograms to predict the risk for severe PPH. This can be used by obstetricians to determine women that are at the highest risk for developing SPPH. The nomograms help patients with RCDs understand their risk of developing SPPH and provides a basis for healthcare providers to identify modifiable risk factors and extend appropriate care to patients at high risk. Future clinical validation of the predictive models or use of other advanced modeling techniques to evaluate the impact of such a nomogram from a public health perspective is warranted. The implementation of these predictive models is expected to improve clinical and public health practice.

## Materials and methods

### Study population

This was a multicenter, large-sample, cross-sectional cohort study conducted in 11 public tertiary hospitals, and it covered 7 provinces, municipalities, and autonomous regions within China (namely Guangdong, Beijing, Xinjiang, Shanxi, Henan, Hubei, and Chongqing). The cohort comprised 14,734 women with scar uterus who delivered again from January 2017 to December 2017 in these 11 hospitals. This retrospective study was approved by the Medical Ethics Committee of Guangzhou Medical University with Medical Research No. 2016 (0406) approved on April 6, 2016. All methods were performed in accordance with the relevant guidelines and regulations. The statements on consent for participation were signed from participants and from legally authorized representatives. Electronic medical records were used to identify women with a history of CD who undergo RCD (including those undergoing elective RCD and those with failure of vaginal birth after cesarean). Pregnancies with < 28 gestational weeks, major fetal abnormalities, antepartum fetal death, multiple pregnancies, myomectomy history, lack of important records, such as delivery mode, amount of blood loss and delivery weeks and severe data loss were excluded. Figure 1 showed a flow diagram and distribution of the patients’ enrollment process.

### Diagnosis

A blood loss of 25% of a woman’s total blood volume (approximately 1,500 mL or more) could lead to tachycardia and hypotension, and is regarded as considerable blood loss^25^. In this study, the criterion for SPPH was an estimated blood loss of 1500 mL or more during RCD or within 24 h after RCD. The blood loss was estimated mainly through gravimetric measurement, namely weight of blood-soaked items subtracting dry weight of the items to obtain blood loss volume. We also collect blood in calibrated canisters through negative pressure aspirator after the birth of the neonate avoiding the problem of measuring nonblood fluids. Endometrial injury other than history of prior cesarean delivery referred to uterine curettage, hysteromyomectomy, hysteroscopic surgery and other reason that damaged the endometrium (A history of a prior cesarean delivery was not included). Uterine incision status during RCD includes normal, thin incision, incomplete and complete rupture.

### Predictive factors

Based on published guidelines and reviews, clinical information related to PPH was collected through chart review in each hospital. This included the general information of patients with RCDs (age, height, weight, nationality, gravida, parity, and history and numbers of abortion), past medical history (numbers of CD, reason for previous CD, interval between the current CD and previous CD, previous history of placenta previa, placenta accreta, etc.), current pregnancy information (assisted reproduction technology, premature rupture of membranes, complicated with placenta previa, etc.). In total, 40 factors were included in Supplementary Table S1 online. Among the 40 factors, 38 factors were pre-operative variables, pelvic condition and uterine scar status during RCD were intraoperative variables.

### Statistical analyses

Statistical analysis was conducted using R software (version 3.6.1), Empower (R) (www.empowerstats.com, X & Y solutions, inc. Boston MA) and SPSS v25.0 for Windows. Patients lack of important records, such as delivery mode, amount of blood loss and delivery weeks and severe data loss were excluded. For other variables the proportion with missing data was listed in Supplementary Table S2 online. Missing values were imputed using Random Forests imputation (predictive mean matching). Quantitative data were examined for normal distribution, non-parametric continuous features were represented as median and interquartile range (IQR) and Mann–Whitney U tests was applied. Categorical variables were reported as frequency (percentage), and the differences between the groups were compared using the χ^2^ test or Fisher exact test in cases of small numbers, as appropriate. The cohort was then divided into the development and the validation cohorts according to the delivery month of patients with RCD (the former 10 months for development set and the latter 2 months for validation set). A comparison for descriptive characteristics in the development and validation cohorts was shown in Supplementary Table S3 online. Collinearity analysis of all variables was done before logistic regression analysis. Variables of strong correlation with outcome in univariate analysis were included in multivariate logistic regression analysis. Multivariable logistic regression models were prepared to estimate the risk of SPPH associated with potential predictors. Independent risk factors were obtained in the development data using logistic backward stepwise selection and model significance test to establish the SPPH prediction model. Four models were established, the first one was a full model using all of the pre-operative and intraoperative variables (40 variables), the second one was a pre-operative simple model using independent risk factors (*P* < 0.05) of the pre-operative variables (38 variables), the third one was an operative simple model using independent risk factors (*P* < 0.05) of the pre-operative and intraoperative variables, the last one was a simple model using variables both in pre-operative and operative simple model. The AUC was used to assess the discrimination of the predictive models. The calibration of the models was evaluated by plotting the calibration curve. Prediction models developed in the development set were validated externally by an assessment of discrimination and calibration in the validation set. Hosmer–Lemeshow was also used to evaluate the models. Different risk cutoff points (2%, 5% and 10%) were used to show the prediction accuracies of the four models. Clinical usefulness and net benefit were estimated with DCA. To provide the clinician with a quantitative tool to predict the individual probability of SPPH, nomograms were developed on the basis of predictive models. Smooth curve fitting, threshold effect and saturation effect analysis between some variables and SPPH were provided (Supplementary Fig. S5 online). The packages in R that were used in this study are reported in the Data Supplement. The reported statistical significance levels were all two-sided, with statistical significance set at 0.05.

### Informed consent

Written informed consent was obtained from the individual participant included in the study.

## Supplementary Information

Supplementary Information 1.

## Data Availability

All data generated or analyzed during this study are included in this published article (and its Supplementary Information files).
