# Supplementary material for: Probability of severe postpartum hemorrhage in repeat cesarean deliveries: a multicenter retrospective study in China
Source: Sci Rep. 2021 Apr 19;11:8434. doi: 10.1038/s41598-021-87830-7 (PMC8055978; doi:10.1038/s41598-021-87830-7)

Probability of severe postpartum hemorrhage in repeat cesarean deliveries: a multicenter retrospective study in China

Lili Du[1,2#], Ling Feng[6#], Shilei Bi[1,2#], Lizi Zhang[8], Jingman Tang[1,2], Liuying Zhong[1,2], Xingnan Zhou[1,2], Hu Tan[1,2], Lijun Huang[1,2], Lin Lin[1,2], Shanshan Zeng[1,2], Luwen Ren[1,2], Yinli Cao[3], Jinping Jia[4], Xianlan Zhao[5], Shaoshuai Wang[6], Xiaoyan Xu[6], Yangyu Zhao[7], Zhijian Wang[8], Qiying Zhu[9], Hongbo Qi[10], Lanzhen Zhang[11], Suiwen Wen[12], Hongtian Li[13], Jingsi Chen[1,2]*, Dunjin Chen[1,2]*

Table S1 The characteristics of the pregnant women in SPPH and non-SPPH group

Variables	Non-SPPH(n=10796)	SPPH(n=278)	P
Age(years)	33(30,36)	34(29,36)	0.807
Height(cm)	160(157,164)	160(156,164)	0.931
Weight(kg)	59(52,65)	60(54,68)	<0.05
Gravida	3(2,3)	3(2,4)	<0.05
Parity	1(1,1)	1(1,2)	<0.05
Numbers of abortion	1(0,1)	1(0,2)	<0.05
Numbers of CD	1(1,1)	1(1,1)	<0.05
Interval months	72(48,108)	72(48,118.5)	0.297
Gestational weeks	39(38,39)	37(35,38)	<0.05
Neonatal birth weight(g)	3235(2940,3500)	2876.5(2437.5,3227.5)	<0.05
Nationality			0.676
Han population	10562(97.8%)	273(98.2%)	
Other	234(2.2%)	5(1.8%)	
Abortion history	6388(59.2%)	206(74.1%)	<0.05

Natural abortion history	1132(10.5%)	39(14%)	0.058
Drug abortion history	725(6.7%)	32(11.5%)	<0.05
Artificial abortion history	5639(52.2%)	185(66.5%)	<0.05
Reason for previous CD			<0.05
Selective	1709(15.8%)	62(22.3%)	
Comorbidity	7010(64.9%)	161(57.9%)	
Other	2077(19.2%)	55(19.8%)	
PP history	138(1.3%)	12(4.3%)	<0.05
Placenta accreta history	19(0.2%)	3(1.1%)	<0.05
Endometrial injury	249(2.3%)	20(7.2%)	<0.05
ART	265(2.5%)	11(4%)	0.113
Source			<0.05
Hospital	8757(81.1%)	187(67.3%)	
Referral	2039(18.9%)	91(32.7%)	
Prom	1422(13.2%)	26(9.4%)	0.062
Vaginal bleeding during pregnancy	894(8.3%)	79(28.4%)	<0.05
Lower uterine segment tenderness	1198(11.1%)	19(6.8%)	<0.05
PP	1247(11.6%)	218(78.4%)	<0.05
Placenta accreta	680(6.3%)	189(68%)	<0.05
PP and placenta accreta	422(3.9%)	176(63.3%)	<0.05

Placenta abruption	114(1.1%)	7(2.5%)	<0.05
Polyhydramnios	747(6.9%)	16(5.8%)	0.449
Oligohydramnios	905(8.4%)	16(5.8%)	0.117
Macrosomia	1723(16%)	8(2.9%)	<0.05
FGR	181(1.7%)	4(1.4%)	1
Hypertension disorders	1183(11%)	30(10.8%)	0.93
GDM	2593(24%)	56(20.1%)	0.135
Type of RCD			0.773
ERCD	10673(98.9%)	276(99.3%)	
Failed TOLAC	123(1.1%)	2(0.7%)	
Reason for CD			<0.05
Selective	6964(64.5%)	111(39.9%)	
Comorbidity	3832(35.5%)	167(60.1%)	
Time of CD			<0.05
Elective	7867(72.9%)	222(79.9%)	
Emergency	2929(27.1%)	56(20.1%)	
Fetal sex			0.52
Male	5996(55.5%)	149(53.6%)	
Female	4800(44.5%)	129(46.45）	
Pelvic adhesion during RCD	2879(26.75）	157(56.5%)	<0.05
Uterine incision status during			
RCD			<0.05

Normal	8135(75.4%)	137(49.3%)
Thin	2513(23.3%)	136(48.9%)
Incomplete rupture	129(1.2%)	4(1.4%)
Rupture	19(0.2%)	1(0.4%)

SPPH: severe postpartum hemorrhage; CD: cesarean delivery; RCD: repeat cesarean delivery; PP: placenta previa; ART: artificial assisted reproductive technology; FGR: fetal growth restriction; GDM: gestational diabetes mellitus; ERCD: elective repeat cesarean delivery; TOLAC: trial of labor after cesarean;

Endometrial injury other than history of prior cesarean delivery includes uterine curettage, hysteromyomectomy, hysteroscopic surgery and other reason that damaged the endometrium

Table S2 Missingness table by group of SPPH

Variables	Non-SPPH(n=10796)	SPPH(n=278)	P
Age(years)	0(0.00%)	0(0.00%)	
Height(cm2)	405(3.75%)	16(5.76%)	0.117
Weight(kg)	864(8.00%)	26(9.35%)	0.48
Gravida	0(0.00%)	0(0.00%)	
Parity	14(0.13%)	1(0.36%)	0.317
Numbers of abortion	0(0.00%)	0(0.00%)	
Numbers of CD	73(0.68%)	2(0.72%)	0.713
Interval months	52(0.48%)	1(0.36%)	1
Gestational weeks	0(0.00%)	0(0.00%)	
Neonatal birth weight(g)	1008(9.34%)	42(15.1%)	0.002
Nationality	22(0.20%)	1(0.36%)	0.443

Abortion history	0(0.00%)	0(0.00%)	
Natural abortion history	300(2.78%)	8(2.88%)	1
Drug abortion history	0(0.00%)	0(0.00%)	
Artificial abortion history	395(3.66%)	11(3.96%)	0.921
Reason for previous CD	0(0.00%)	0(0.00%)	
PP history	851(7.88%)	22(7.91%)	1
Placenta accreta history	1368(12.7%)	52(18.7%)	0.004
Endometrial injury	1586(14.7%)	27(9.71%)	0.025
ART	57(0.53%)	5(1.8%)	0.019
Source	56(0.52%)	0(0.00%)	0.406
Prom	546(5.06%)	12(4.32%)	0.675
Vaginal bleeding during pregnancy	611(5.66%)	12(4.32%)	0.408
Lower uterine segment tenderness	618(5.72%)	16(5.76%)	1
PP	645(5.97%)	9(3.24%)	0.075
Placenta accreta	671(6.22%)	9(3.24%)	0.055
PP and placenta accreta	650(6.02%)	8(2.88%)	0.039
Placenta abruption	695(6.44%)	16(5.76%)	0.738
Polyhydramnios	689(6.38%)	15(5.4%)	0.588
Oligohydramnios	680(6.3%)	17(6.12%)	1
Macrosomia	763(7.07%)	17(6.12%)	0.621
FGR	703(6.51%)	18(6.47%)	1
Hypertension disorders	665(6.16%)	18(6.47%)	0.929

GDM	636(5.89%)	18(6.47%)	0.78
Type of RCD	0(0.00%)	0(0.00%)	
Reason for RCD	1405(13.0%)	44(15.8%)	0.199
Fetal sex	0(0.00%)	0(0.00%)	
Pelvic adhesion during RCD	805(7.46%)	21(7.55%)	1
Uterine incision status during RCD	857(7.94%)	26(9.35%)	0.455

SPPH: severe postpartum hemorrhage; CD: cesarean delivery; RCD: repeat cesarean delivery PP: placenta previa; ART: artificial assisted reproductive technology; FGR: fetal growth restriction; GDM: gestational diabetes mellitus;

Endometrial injury other than history of prior cesarean delivery includes uterine curettage, hysteromyomectomy, hysteroscopic surgery and other reason that damaged the endometrium

Table S3 The characteristics of the pregnant women in development and validation group

Variables	Development(n=9177)	Validation(n=1897)	P
Age(years)	33(30,36)	34(30,36)	<0.05
Height(cm)	160(157,164)	160(157,164)	0.42
Weight(kg)	59(52,66)	57(52,64.5)	<0.05
Gravida	3(2,3)	3(2,4)	<0.05
Parity	1(1,1)	1(1,1)	<0.05
Numbers of abortion	1(0,1)	1(0,1)	0.107
Numbers of CD	1(1,1)	1(1,1)	<0.05
Interval months	72(48,108)	66(48,105)	<0.05
Gestational weeks	39(38,39)	39(38,39)	<0.05

Neonatal birth weight(g)	3250(2950,3510)	3150(2850,3450)	<0.05
Nationality			<0.05
Han population	8942(97.4%)	1893(99.8%)	
Other	235(2.6%)	4(0.2%)	
Abortion history	5477(59.7%)	1117(58.9%)	0.518
Natural abortion history	957(10.5%)	204(10.8%)	0.78
Drug abortion history	558(6.1%)	199(10.5%)	<0.05
Artificial abortion history	4879(53.2%)	945(49.8%)	<0.05
Reason for previous CD			<0.05
Selective	1646(17.9%)	125(6.6%)	
Comorbidity	5844(63.7%)	1327(70.0%)	
Other	1687(18.4%)	445(23.5%)	
PP history	123(1.3%)	27(1.4%)	0.776
Placenta accreta history	15(0.2%)	7(0.4%)	0.067
Endometrial injury	216(2.4%)	53(2.8%)	0.257
ART	237(2.6%)	39(2.1%)	0.180
Source			<0.05
Hospital	7902(86.1%)	1042(54.9%)	
Referral	1275(13.9%)	855(45.1%)	
Prom	1266(13.8%)	182(9.6%)	<0.05
Vaginal bleeding during pregnancy	845(9.2%)	128(6.7%)	<0.05

Lower uterine segment			
tenderness	1119(12.2%)	98(5.2%)	<0.05
PP	1255(13.7%)	210(11.1%)	<0.05
Placenta accreta	701(7.6%)	168(8.9%)	0.073
PP and placenta accreta	460(5.0%)	138(7.3%)	<0.05
Placenta abruption	112(1.2%)	9(0.5%)	<0.05
Polyhydramnios	701(7.6%)	62(3.3%)	<0.05
Oligohydramnios	815(8.9%)	106(5.6%)	<0.05
Macrosomia	1070(11.7%)	661(34.8%)	<0.05
FGR	138(1.5%)	47(2.5%)	<0.05
Hypertension disorders	1038(11.3%)	175(9.2%)	<0.05
GDM	2289(24.9%)	360(19.0%)	<0.05
Type of RCD			<0.05
ERCD	9082(99.0%)	1867(98.4%)	
Failed TOLAC	95(1.0%)	30(1.6%)	
Reason for CD			<0.05
Selective	6220(67.8%)	855(45.1%)	
Comorbidity	2957(32.2%)	1042(54.9%)	
Time of CD			<0.05
Elective	6649(72.5%)	1440(79.9%)	
Emergency	2528(27.5%)	457(24.1%)	
Fetal sex			<0.05

Male	5044(55.0%)	1101(58.0%)	
Female	4133(45.0%)	796(42.0%)	
Pelvic adhesion during RCD	2595(28.3%)	441(23.2%)	<0.05
Uterine incision status during RCD			<0.05
Normal	6864(74.8%)	1408(74.2%)	
Thin	2164(23.6%)	485(25.6%)	
Incomplete rupture	129(1.4%)	4(0.2%)	
Rupture	20(0.2%)	0(0.0%)	

SPPH: severe postpartum hemorrhage; CD: cesarean delivery; RCD: repeat cesarean delivery; PP: placenta previa; ART: artificial assisted reproductive technology; FGR: fetal growth restriction; GDM: gestational diabetes mellitus; ERCD: elective repeat cesarean delivery; TOLAC: trial of labor after cesarean;

Endometrial injury other than history of prior cesarean delivery includes uterine curettage, hysteromyomectomy, hysteroscopic surgery and other reason that damaged the endometrium

Fig. S1 Distributions of SPPH Probabilities.

The distribution of SPPH probabilities, based on the simple model, is shown among persons with SPPH and those without SPPH in the development and Validation data sets.

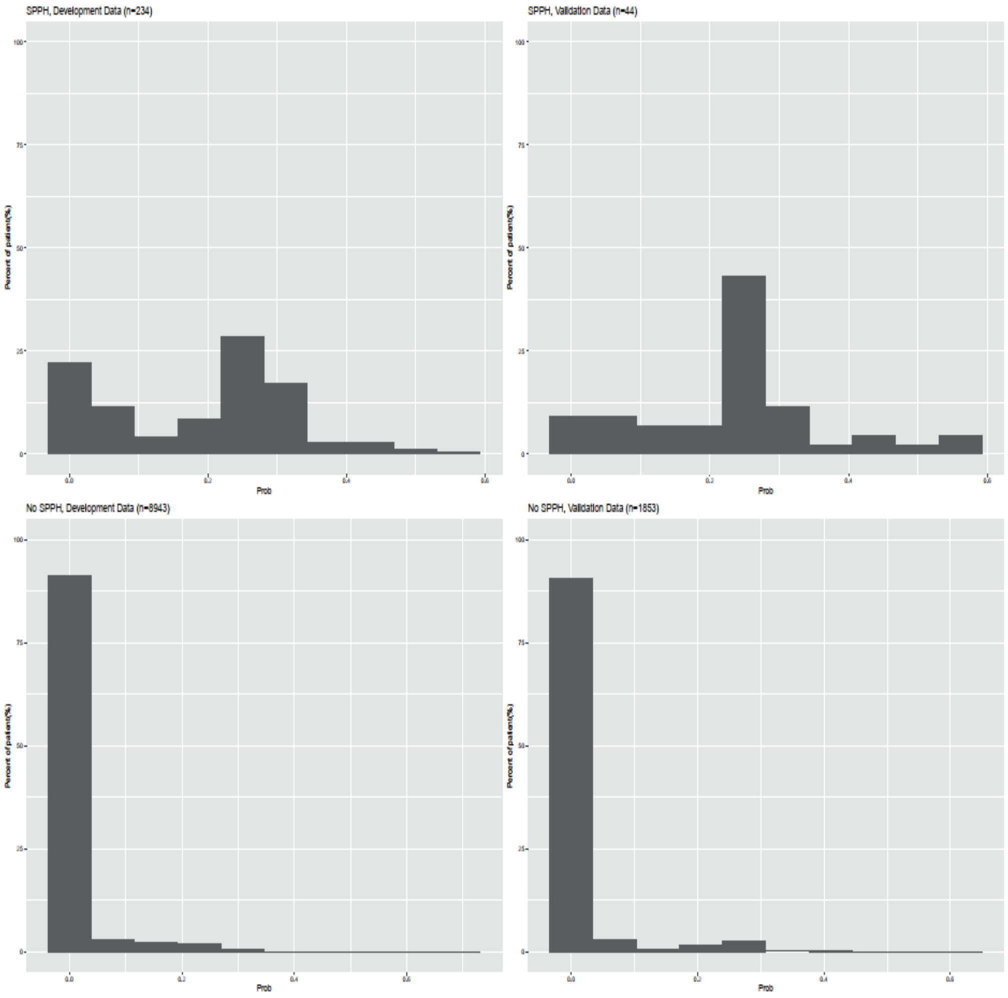

Fig. S2 DCA of the four models in development data and validation data

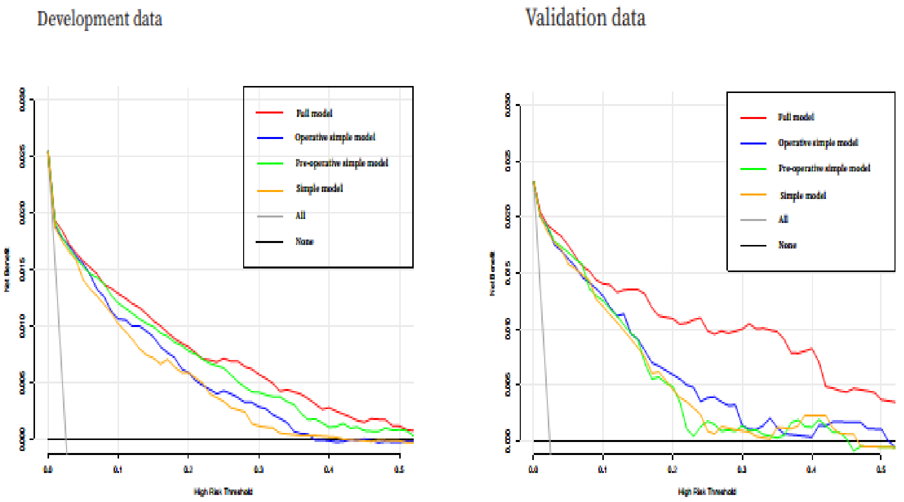

Fig. S3 Nomogram of pre-operative model.

Caption: CD: cesarean delivery; PP: placenta previa; SPPH: severe postpartum hemorrhage;

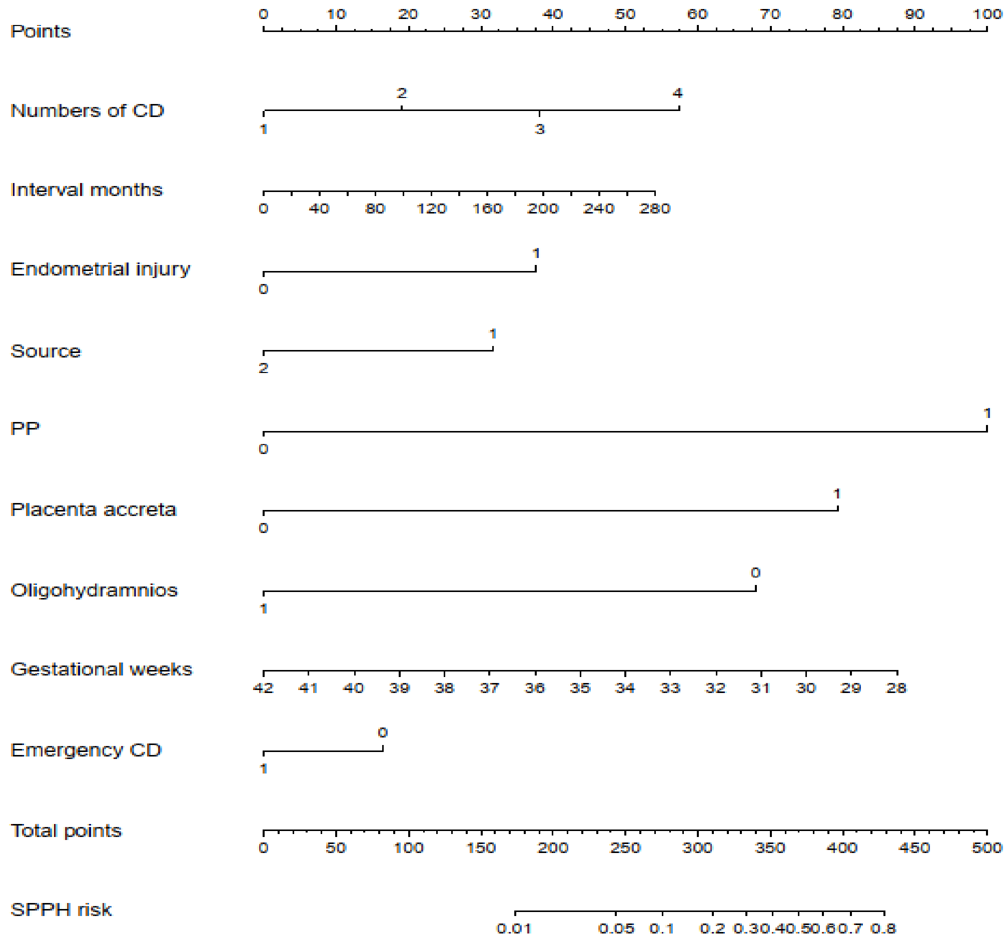

Fig. S4 ROC curves based on the simple model in three separate hospital data sets.

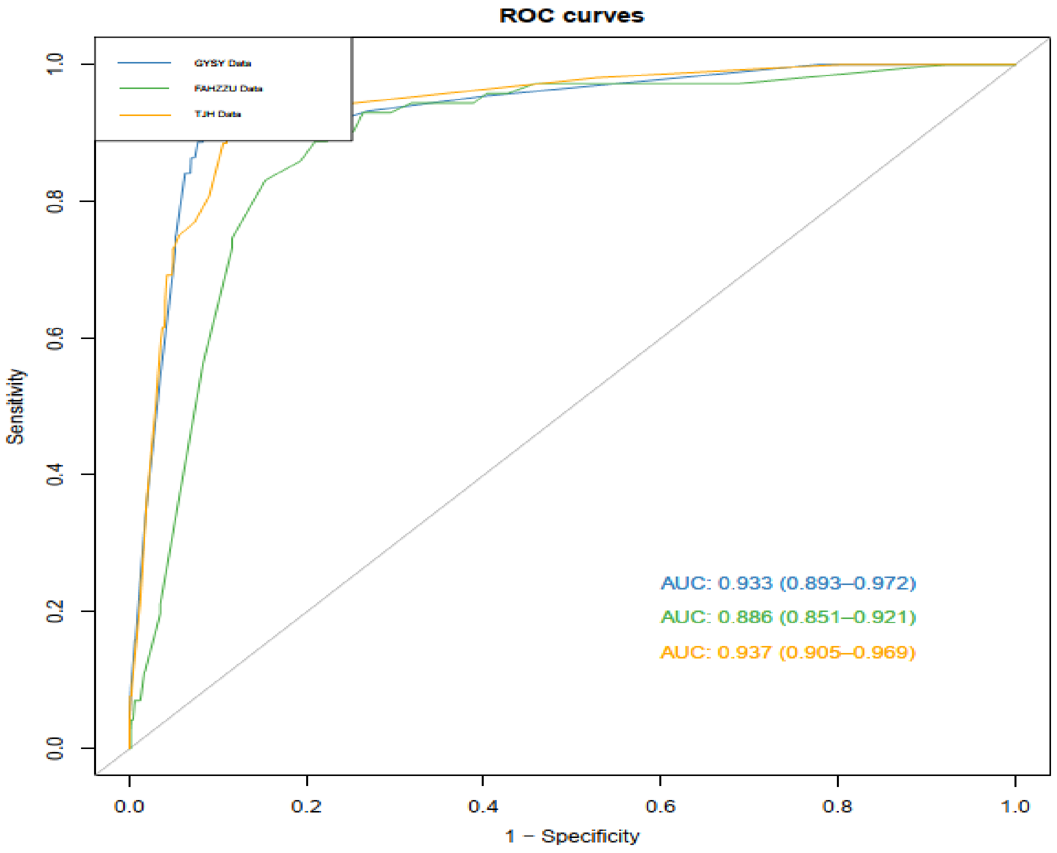

Fig. S5 A: Smooth curve fitting of interval month and SPPH; B: Smooth curve fitting of gestational week and SPPH

A

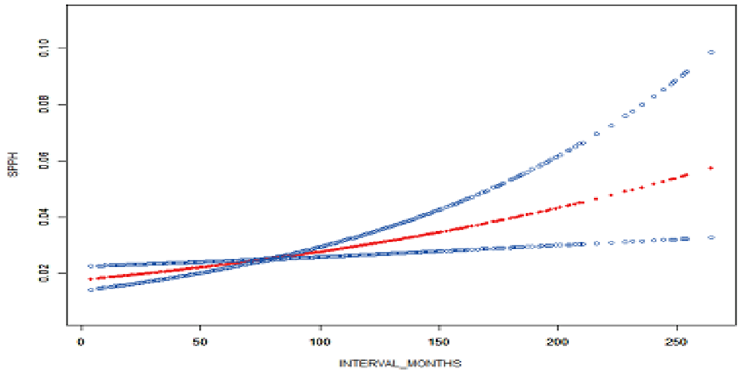

B

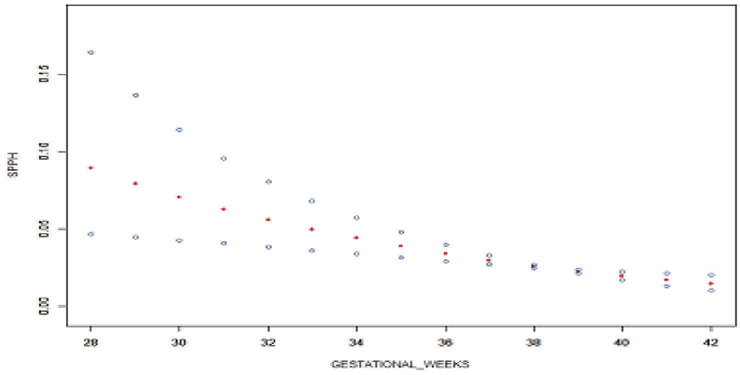

Supplement: Supplementary file 1 — Supplementary Information 1. [file 41598_2021_87830_MOESM1_ESM.pdf]
